# Favorable response to doxorubicin combination chemotherapy does not yield good clinical outcome in patients with metastatic breast cancer with triple-negative phenotype

**DOI:** 10.1186/1471-2407-10-527

**Published:** 2010-10-05

**Authors:** Seong Yoon Yi, Jin Seok Ahn, Ji Eun Uhm, Do Hyoung Lim, Sang Hoon Ji, Hyun Jung Jun, Kyoung Ha Kim, Myung Hee Chang, Min Jae Park, Eun Yoon Cho, Yoon La Choi, Yeon Hee Park, Young-Hyuck Im

**Affiliations:** 1 Division of Hematology-Oncology, Department of Medicine, Samsung Medical Center, Sungkyunkwan University School of Medicine, 50 Irwon-dong Gangnam-gu, Seoul 135-710 Korea; 2 Department of Pathology, Samsung Medical Center, Sungkyunkwan University School of Medicine, 50 Irwon-dong Gangnam-gu, Seoul 135-710 Korea

## Abstract

**Background:**

We analyzed the responses to first line treatment and clinical outcomes of metastatic breast cancer patients treated with palliative doxorubicin/cyclophosphamide (AC) according to molecular cancer subtype.

**Methods:**

A retrospective analysis was performed for 110 metastatic breast cancer patients selected on the basis of palliative AC treatment and the availability of immunohistochemical data for estrogen receptor (ER), progesterone receptor (PR), and human epidermal growth factor receptor-2 (HER-2/neu) status.

**Results:**

Of the 110 patients analyzed, 71 (64.5%) were hormone receptor positive (HR+), 14 (12.7%) were HER2+, and 25 (22.7%) were triple negative (TN). There were no differences in age, stage at diagnosis, total number of cycles of palliative chemotherapy, incidence of visceral metastasis, and metastatic sites with the exception of liver among breast cancer subtypes. The overall response rates to AC were 55.9% for the HR+ subgroup, 42.9% for the HER2+ subgroup, and 56.5% for the TN subgroup. The progression-free survival (PFS) in patients with HER2+ and TN were significantly shorter than in the HR+ (median PFS, 9.1 *vs *8.1 *vs *11.5 months, respectively; p = 0.0002). The overall survival (OS) was 25.4 months in the TN subgroup and 27.3 months in HER2+ subgroup. The median OS for these two groups was significantly shorter than for patients in the HR+ subgroup (median, 38.5 months; 95% CI, 30.1-46.9 months; p < 0.0001).

**Conclusions:**

The response to palliative AC chemotherapy did not differ among breast cancer subtypes. Despite chemosensitivity for palliative AC, the TN subtype has a shorter overall survival than non-TN subtypes. Innovative treatment strategies should be developed to slow the course of disease.

## Background

Breast cancer encompasses a heterogeneous group of diseases at the molecular level [1-4], and can be classified into at least five distinct subtypes by gene expression profiling: luminal A, luminal B, normal breast-like, ERBB2, and basal like [5-10]. Basal cell-like tumors typically show low or no expression of HER2 and estrogen receptor (ER), and high expression of genes characteristic of basal epithelial cells [1,4,11-13]. These tumors may share clinical and biologic properties with triple negative breast cancers (TNBCs) that lack expression of ER, progesterone receptor (PgR) and HER2 [5-8]. There is increasing evidence that breast cancer molecular subtypes differ in their responses to therapeutic agents. TNBC is considered a high-risk subtype because of its typically younger patient age, poorly differentiated tumor characteristics, and shortened survival of patients who often do not benefit from targeted therapies [11-19]. Because there is insufficient data on which to base treatment selection, no specific systemic treatment strategy is currently recommended for the treatment of TNBC.

Anthracyclines are some of the most widely used and effective drugs to treat patients with breast cancer in the adjuvant setting, as well as for patients with metastatic disease [20-25]. However, cumulative cardiotoxicity is a major limitation to the therapeutic use of anthracyclines and can lead to potentially fatal congestive heart failure [26-28]. Nevertheless anthracyclines are still the most widely used chemotherapeutic agents for the treatment of breast cancer. Because of their wide use and proven efficacy, anthracyclines may be especially important for the treatment of TNBCs that are known to lack specific therapeutic targets. Doxorubicin shows pre-clinical and clinical activity against BRCA1-associated cancers, a closely related group of diseases with significant morphologic, phenotypic, and genetic overlap with TNBCs [29,30]. This further suggests the potential of anthracycline chemotherapy as a therapeutic option for TNBCs.

Given the interest in using doxorubicin for treating patients with TN tumors, we have retrospectively reviewed the medical records of metastatic breast cancer patients who received combination chemotherapy with doxorubicin and cyclophosphamide (AC) as a first line treatment. The aim of this study was to evaluate the efficacy of AC combination therapy in patients with TNBC. In addition, we characterized the clinicopathologic findings relating to the response to AC chemotherapy.

## Methods

### Patients

We performed a retrospective analysis of medical records of patients with metastatic or recurrent breast cancer who received AC combination chemotherapy as a first-line treatment at Samsung Medical Center between January 2001 and December 2006. These studies were approved by the Samsung Medical Center Institutional Review Board.

### Immunohistochemistry and molecular classification

All pathologic specimens were reviewed by two experienced pathologists who determined the status of ER, PgR, and HER2 using immunohistochemical (IHC) techniques. According to the Allred scoring system, ER and PgR negativity was defined as a total score from 0-2 by IHC using antibodies to the ER (Immunotech, France) and PgR (Novocastra, UK). According to National Comprehensive Cancer Network (NCCN) guidelines, HER2 was assessed using IHC techniques (DAKO, Santa Barbara, CA, USA) and/or fluorescence *in situ *hybridization (FISH). IHC grades 0 and 1 were defined as a negative result for HER2, and the lack of HER2 amplification was confirmed by FISH if HER2 was rated 2+ by IHC. "Triple negativity" was defined as a lack of expression of ER, PgR, and HER2. The HER2+ subtype was defined as HER2-positive with ER- and PR-negative, while hormone receptor-positive was defined as ER- and/or PR-positive, regardless of HER2-positivity subtypes. Ki-67 growth fractions and p53 status were assessed using antibodies: Ki-67 (DACO, Glostrup, Denmark), and p53 (1:80, Zymed, San Francisco, CA, USA). The percentage of positive nuclei stained for Ki-67 was calculated for each section based on approximately 1,000 carcinoma cell nuclei. High proliferative index was defined as 50% or more stained nuclei. Immunoreactivity of p53 was defined as greater than 5% of cells having distinct nuclear staining.

### Statistical analysis

The treatment response to AC was evaluated by reviewing imaging studies according to the Response Evaluation Criteria in Solid Tumors (RECIST). AC chemotherapy was doxorubicin 60 mg/m^2 ^plus cyclophosphamide 600 mg/m^2 ^every 3 weeks. Progression-free survival (PFS) was defined as the time from the initial date of AC chemotherapy to the date that progressive disease was first documented. Overall survival (OS) was defined as the time from the initial date of AC chemotherapy to the date of death from any cause, or the date of last follow-up. The calculation was performed using the Kaplan-Meier method. The log-rank method (also known as the Mantel-Cox test) was used to compare survival rates. The differences in responses between subtypes were estimated by the χ^2 ^test or Fisher's exact test. A p < 0.05 was considered significant.

## Results

### Patient cohort

Between January 2001 and December 2006, 124 patients with metastatic breast cancer were treated with palliative AC as a first-line treatment at Samsung Medical Center. Immunohistochemical data for ER, PgR, and HER2 status were available for 110 of these patients. Among them, sixty-eight patients relapsed after surgery for invasive breast cancers, and forty-two patients had metastatic diseases at diagnoses.

Subgroups were classified on the basis of ER, PR, and HER2 IHC findings with 71 of the 110 patients (64.5%) being HR+, 14 patients (12.7%) HER2+, and 25 patients (22.7%) TN. The median age at diagnosis was 45 years (range, 24-69 years). Median disease free intervals (DFI) after surgery among relapsed patients were 47.6 months for HR+, 40.6 months for HER2+, and 38.7 months for TNBC (p = 0.231). Forty patients (36.4%) were initially diagnosed with metastatic disease. TNBCs showed poorer histologic differentiation (Bloom-Richardson pathologic grade III: 87.5% for TNBC, 25.0% for HER2+, and 37.8% for HR+, respectively; p = 0.001) and a higher proliferative index (Ki-67 >50%: 85.7% for TNBC, 0% for HER2+, and 31.3% for HR+, respectively; p = 0.013) than HER2+ or HR+ subtypes. Other clinical features, such as age, stage at diagnosis, and previous treatment did not differ significantly among the three subtypes. Adjuvant doxorubicin was administered to 7 patients (6 in HR+, 1 in HER2+). Hepatic metastases were more common in the HER2+ subgroup (52.4% for HR+, 84.6% for HER2+, 45.5% for TNBC; p = 0.046). Adjuvant endocrine treatment was performed for 43 HR+ patients (Table 1). Next to AC chemotherapy, median numbers of palliative chemotherapy regimens were 3 for HR+, 2 for HER2+, and 1 for TNBC (p = 0.006). Endocrine treatment was treated for 17 HR+ patients (24%) as palliative aim (Table 1).

**Table 1:** Clinicopathologic characteristics of patients according to subtypes

	HR+ (N = 71)	HER2+ (N = 14)	TN (N = 25)	*P value*
**Clinical characteristics at initial diagnosis**				
Median age	43.8 (30-69)	49.5 (33-65)	47.0 (24-77)	*.398*^*A*^
Initial stage				
I	9 (12.7%)	1 (7.1%)	2 (8.0%)	
II	23 (32.4%)	4 (28.6%)	11 (44.0%)	
III	11 (15.5%)	2 (14.3%)	2 (8.0%)	
IV	25 (35.2%)	6 (42.9%)	9 (36.0%)	*.951*^*B*^
unknown	3 (4.2%)	1 (7.1%)	1 (4.0%)	
Familial History	16 (23.5%)	4 (28.6%)	7 (29.2%)	*.829*^*B*^
DFI after surgery (median)	47.6 months	40.6 months	38.7 months	*0.231 by log-rank test*
**Tumor characteristics**				
B-R.Gr. III (n = 69)	17 (37.8%)	2 (25.0%)	14 (87.5%)	*.001^***B***^
P53 <5% (n = 93)	32 (55.2%)	7 (50.0%)	9 (42.9%)	*.621*^*B*^
Ki 67 >50% (n = 25)	5 (31.3%)	0 (0%)	6 (85.7%)	*.013^***B***^
**Previous (neoadjuvant or adjuvant) treatments**				
Curative surgery.	45 (63.4%)	8 (57.1%)	15 (60.0%)	*.888*^*B*^
Neoadju-CTx	3 (4.2%)	0 (0%)	1 (4.0%)	*.573*^*B*^
Adjuvant CTx	35 (49.3%)	7 (50.0%)	12 (48.0%)	*.991*^*B*^
Adjuvant doxorubicin	6 (17.1%)	1 (14.3%)	0 (0%)	*.048*^*B*^
Adjuvant HormoTx	43 (60.6%)	0	0	*< 0.0001 *^*B*^
**Distant Metastases**				
Lung	49 (74.2%)	11 (84.6%)	15 (62.5%)	*0.316*
Pleura	41 (57.8%)	8 (57.4%)	10 (45.5%)	0.216
Liver	33 (52.4%)	11 (84.6%)	10 (45.5%)	*0.046*
Brain	11 (17.7%)	3 (23.1%)	6 (27.3%)	0.629
Bone	36 (50.7%)	8 (57.1%)	10 (40.0%)	*0.283*
Skin	14 (19.7%)	1 (7.1%)	9 (36%)	*0.081*
Soft tissue	22 (31.0%)	1 (7.1%)	6 (24.0%)	0.120
**Next treatment after AC chemotherapy**				
Median number of CTx (range)	3 (0-11)	2 (0-8)	1 (0-7)	*0.006*
Number of patients with HormoTx (%)	17 (24%)	0	0	*0.001*
Number of patients with trastuzumab Tx (%)	5 (7.0%)	4 (28.6%)	0	*0.009*

DFI, disease free interval; CTx, chemotherapy; HormoTx, hormonal therapy; Tx, treatment

^A^Continuous variables; One way ANOVA

^B^Categorical variables; Pearson's chi-square test

### Treatment responses

Of the 110 patients, 105 (95%) with measurable lesions had a clinical response. Two of these 105 patients had a complete response (CR), and 56 patients achieved a partial response (PR; Table 2). Both patients with CR were HR+. The overall response rate (ORR) of 105 patients with measurable disease was 54.3%. The ORR of HER2+ patients was slightly lower than those of patients with HR+ or TN-subtypes, but this difference was not statistically significant (42.9%, 55.9%, and 56.5%, respectively; p = 0.542; Table 2).

**Table 2:** Treatment Response According to Subtypes (n = 105 patients with measurable diseases)

	Total (n = 105)	HR (n = 68)	HER2 (n = 14)	TN (n = 23)	*p*
Median cycles	6.0 (1-12)	5.9 (1-12)	4.8 (1-9)	5.6 (1-9)	0.454
Response					
CR	2	2	0	0	
PR	55	36	6	13	
SD	25	19	3	4	
PD	20	11	4	5	
Not evaluable	2	0	1	1	
Overall response rate	57 (54.3%)	38 (55.9%)	6 (42.9%)	13 (56.5%)	0.542

CR; complete response, PR; partial response, SD; stable disease, PD; progressive disease

### Survival analysis

#### Progression-free survival (PFS)

**Figure 1:**
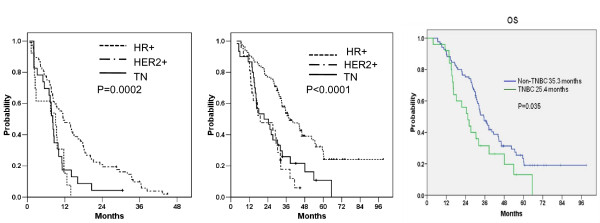
**Kaplan-Meier survival curves according to subtypes**. A Progression-Free Survival (PFS) to AC chemotherapy. B Overall Survival (OS) to AC chemotherapy according to subgroups. C Overall Survival (OS) to AC chemotherapy in patients with TNBCs and non-TNBCs

The median PFS to AC chemotherapy was 9.5 months (95% CI, 7.1-11.9 months). The median PFS of each phenotype was 8.1 months for TN (95% CI, 7.2-9.1 months), 9.1 months for HER2+ (95% CI, 1.3-16.8 months), and 11.5 months for HR+ (95% CI, 7.2-15.9 months; p = 0.0002 by log-rank test; Figure 1A).

#### Overall survival

With a median 55.8 months of follow-up (range, 29.2-101.5 months), the median OS of all 110 patients was 32.4 months (95% CI, 28.5-36.3 months). The median OS was 25.4 months (95% CI, 17.9-32.9 months) in the TN subgroup and 27.3 months (95% CI, 9.3-45.3 months) in the HER2+ subgroup. The median OS for these two groups was significantly shorter than OS for HR+ patients (median, 38.5 months; 95% CI, 30.3-46.7 months; p < 0.0001). Most of the HER2+ patients did not receive trastuzumab as first-line treatment with chemotherapy (Figure 1B).

#### Time-to-Death (TTD) from the end of AC chemotherapy

There is a significant difference between patients with TN and non-TN phenotypes in terms of TTD after AC chemotherapy (median TTD 15.9 months, 95% CI, 7.8-23.9 months for TN; median TTD 13.2 months, 95% CI, 9.7-16.7 months for HER2+; median TTD 24.8 months; 95% CI, 21.7-36.70 months for HR+; p < 0.0001). After AC chemotherapy, most of the patients were treated with additional 2^nd ^or more lines of chemotherapies with various regimens including platinum and taxane agents. A median of three additional lines of palliative chemotherapies were undertaken for HR+ patients, whereas a median of two lines and one line of chemotherapies were performed for HER2+ and TNBC patients, respectively (p = 0.006). 17 of 71 (24%) of HR+ patients were treated with palliative endocrine treatment. 9 of 14 HER2+ patients received palliative trastuzumab treatment with or without other cytotoxic agents.

#### Multivariate analysis for survival

The TN phenotype and HER2 positivity were identified as independent prognostic factors for survival in Cox-regression multivariate analysis (hazard ratio [HR] 2.03, p = 0.011 for TN; HR 1.86, p = 0.027 for HER2 positivity) (Table 3).

**Table 3:** Multivariate Cox-regression analysis on metastatic OS

	P value	Hazard Ratio (HR)	95% CI	
				Triple negativity	**0.011**	**2.03**	1.17	3.51
				HER2 positivity	**0.027**	**1.86**	1.07	3.22

OS: overall survival, 95% CI: 95% confidence interval

## Discussion

The present study demonstrates that TNBC has a similar response rate to AC combination chemotherapy compared to other subtypes (Table 2). This encouraging result is in agreement with published data regarding chemotherapy for TNBCs, irrespective of the chemotherapy regimen [31-33]. Despite the initial sensitivity to chemotherapy, several studies have shown that TNBCs carry a poor prognosis [11-13]. Our results also showed that TNBCs had poorer PFS and OS than non-TNBCs (Figure 1C). This finding suggests that despite the beneficial response to treatment, chemotherapy did not improve subsequent long-term survival of TNBC patients. Several studies support this paradoxical feature of high response, but poor long-term outcome [3,4,32-34].

Chemotherapy responsiveness is usually positively correlated with good clinical outcomes resulting in long-term survival [35]. However, this chemosensitivity is insufficient to overcome biologic aggressiveness, and rapid tumor-growth characteristics of TNBCs. In fact, the TTD after AC chemotherapy in patients with TNBCs was much shorter than that for HR+ patients (median TTD, 15.9 months for TN, 13.2 months for HER2+, and 24.8 months for HR+; p = 0.0037). This finding suggests that another therapeutic strategy is needed to overcome the aggressive clinical behavior, prolong the survival time and improve the quality of life of TNBC patients. In contrast, a longer TTD after AC chemotherapy in patients with non-TNBCs, mainly of the HR+ subtype, may be due to the relatively slower tumor growth characteristics and additional benefit from hormonal therapies with subsequent easier disease control than for TNBCs. Thus, future treatment strategies should be developed based on the heterogeneity of the tumor. The consideration of such tumor heterogeneity will make a valuable contribution to the design of individualized therapy.

Dose intensification of conventional chemotherapeutic agents including doxorubicin may be beneficial to extend the therapeutic index [36]. However, most of the patients did not have durable responses. This suggests that doxorubicin resistance can easily occur in metastatic TNBC, perhaps due to prior exposure to doxorubicin or other chemotherapeutic agents in the adjuvant setting. Additionally, considering the poor correlation between chemosensitivity and long-term survival in patients with TNBCs, it is necessary to substantially prolong the response duration. This is because once lack of response occurs, TNBCs appears to follow a more aggressive and fatal clinical course TNBCs have more aggressive tumor biology as evidenced by a higher histological grade and higher expression of Ki-67 than non-TNBCs. Recently, a few reports have suggested the potential use of these proliferation indexes as makers for complex tumor characteristics [33,37,38]. Importantly, TNBCs are composed of heterogeneous disease entities, consisting of two or more subtypes [39,40].

The interpretation of our study may be limited, because it is a retrospective study. In addition, prior adjuvant therapy may affect on clinical outcomes in a metastatic setting. Despite these drawbacks, our study may have clinical impact in treating TNBC, for which therapeutic options are limited. The conventional treatments studies here need to be supplemented with new innovative therapeutic strategies based on an understanding of the biologic differences and aggressiveness of TNBCs. As HER2 direct therapy in HER2 positive subtype, new innovative targets for TNBC need to be developed. Ongoing clinical trials may open the door to better treatment outcomes. These trials evaluating new drugs such as PARP1 inhibitors [40-42], EGFR tyrosine kinase inhibitors [43], and antiangiogenic agents for TNBCs in adjuvant and palliative settings.

## Conclusions

TNBC had a comparable response rate to first-line AC chemotherapy compared to non-TNBC. Nonetheless, TNBC patients had shorter survival than non-TNBC patients. Innovative therapeutic strategies are needed to overcome the biologic aggressiveness of TNBCs.

## Competing interests

The authors declare that they have no competing interests.

## Authors' contributions

SYY carried out raw data and primary statistical analyses. JSA planned basic design of the study and coordination. JEU, DHL, and SHJ participated in data analysis and draft writing. HJJ, KHK, MHC, and MJP participated in clinical data collection and assisted statistical analysis. EYC and YLC carried out the immunohistochemical staining and evaluated the immunostaining. YHP participated in writing the manuscript and coordination. YHI organized and supervised the study. All authors read and approved the final manuscript.

## Pre-publication history

The pre-publication history for this paper can be accessed here:

http://www.biomedcentral.com/1471-2407/10/527/prepub
